# Supplementary material for: The Effect of n-3 PUFA Binding Phosphatidylglycerol on Metabolic Syndrome-Related Parameters and n-3 PUFA Accretion in Diabetic/Obese KK-Ay Mice
Source: Nutrients. 2019 Nov 22;11(12):2866. doi: 10.3390/nu11122866 (PMC6950267; doi:10.3390/nu11122866)
Supplement: Supplementary file 1 [file nutrients-11-02866-s001.pdf]

Supplementary Material

Supplemental Table S1. Nutrient of the experimental diets

Diet Ingredient (g/kg Diet)	Diets		
	SoyPC	n-3 PUFA–PG	n-3 PUFA–TAG
Cornstarch	397.86	397.86	397.86
Dextrinized Starch	132	132	132
Casein	200	200	200
Sucrose	100	100	100
Cellulose	50	50	50
Soybean Oil	50.000	50.000	56.568
SoyPC	20	-	-
n-3 PUFA-PG	-	20	-
n-3 PUFA-TAG*	-	-	13.432
(DHA-55-TAG)	-	-	(5.358)
(EPA-28-TAG)	-	-	(8.074)
L-Cystine	3	3	3
AIN-93G Mineral Mixture	35	35	35
AIN-93G Vitamin Mixture	10	10	10
Choline Bitartrate	2.5	2.5	2.5
tert-Butylhydroquinone	0.014	0.014	0.014

SoyPC, soybean phosphatidylcholine; n-3 PUFA-PG, n-3 polyunsaturated fatty acid enriched phosphatidylglycerol; n-3 PUFA-TAG, n-3 polyunsaturated fatty acid enriched triacylglycerol. * n-3 PUFA-TAG was prepared by mixing with DHA-55-TAG and EPA-28-TAG (5.358:8.074, w/w)

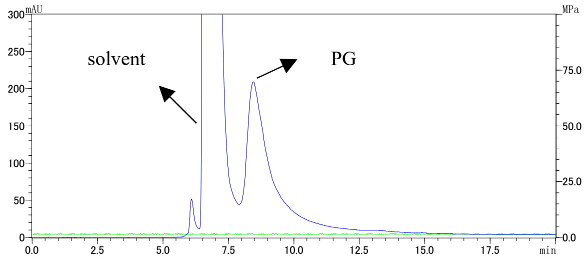

Supplemental Figure. S1 Chromatography of prepared n-3 PUFA-PG. Phospholipids were measured using a high-performance chromatography (HPLC) system equipped with a diode array detector at 210 nm. Mightysil Si60 column (5 μm, 4.6 mm × 250 mm) was connected for the analysis. The mobile phase was acetonitrile/methanol (98:2, v/v) containing 0.5% (v/v) sulfuric acid and eluted at the flow rate of 1.0 mL/min for 20 min, the sample injection was 10 μL.
